# Hemin Attenuates Cisplatin-Induced Acute Renal Injury in Male Rats

**DOI:** 10.1155/2014/476430

**Published:** 2014-09-22

**Authors:** Mohamed A. Al-Kahtani, Ashraf M. Abdel-Moneim, Omar M. Elmenshawy, Mohamed A. El-Kersh

**Affiliations:** ^1^Department of Biological Sciences, Faculty of Science, King Faisal University, Al-Hassa 31982, Saudi Arabia; ^2^Department of Zoology, Faculty of Science, Alexandria University, Alexandria 21511, Egypt; ^3^Department of Zoology, Faculty of Science, Al-Azhar University, Nasr City 11884, Egypt; ^4^Department of Chemistry, Faculty of Science, King Faisal University, Al-Hassa 31982, Saudi Arabia; ^5^Department of Biochemistry, Faculty of Science, Alexandria University, Alexandria 21511, Egypt

## Abstract

*Background.* The aim of this study is to investigate the protective effects of hemin (the heme oxygenase-1 [OH-1] inducer) against nephrotoxic effects induced by cisplatin [cis-diamminedichloroplatinum II (CP)] in male rats. *Methods.* The evaluation was performed through monitoring renal redox parameters: lipid peroxidation (LPO), glutathione peroxidase (GPx), superoxide dismutase (SOD), glutathione reductase (GR), and reduced glutathione (GSH). The work also examined renal function tests (urea and creatinine), tissue proinflammatory mediator like nitric oxide (NO), and kidney cytopathology. *Results.* A single intraperitoneal dose of CP (10 mg/kg b.w.) caused significant elevation of blood urea, serum creatinine, and renal LPO and NO, along with significant decline of the activities of GPx and GR, but renal SOD activity and GSH level were statistically insignificant as compared to control group. Subcutaneous injection of hemin (40 *µ*mol/kg b.w.) partially ameliorated CP-induced renal damage, based on suppression of blood urea, serum creatinine, the renal MDA and NO levels, and increased antioxidant capacity in CP-treated rats. The results of histopathological and ultrastructural investigations supported the renoprotective effect of hemin against CP-induced acute toxicity. *Conclusion.* The induction of HO-1 by hemin is a promising approach in the treatment of CP-induced nephrotoxicity. However, further preclinical studies are warranted to test effectiveness of CP/hemin on the outcome of tumor chemotherapy.

## 1. Introduction

Cisplatin (cis-diamminedichloroplatinum(II), CP) is a highly effective chemotherapeutic agent against a large spectrum of tumor types [1–3]. However, the long-term clinical use of CP is limited by its serious side effects, mainly nephrotoxicity [4]. CP causes impairment of kidney function and acute renal failure via multiple mechanisms including generation of oxygen/nitrogen species, DNA damage, tubulointerstitial inflammation, and apoptotic cell death [5–12]. A number of studies have evaluated compounds as potential nephroprotectors against CP; these included natural antioxidants, modulators of nitric oxide synthesis, osmotic diuretics, and cytoprotective and antiapoptotic agents [13]. However, most of them were not found suitable/safe for clinical practice. In this context, heme oxygenase-1 (HO-1), the rate-limiting enzyme in heme catabolism, might offer a promising alternative. HO-1 (also known as heat shock protein 32) is induced by free radical-initiated reactions, and its induction is considered to be an adaptive response against oxidative tissue damage [14–20]. In addition, HO-1 has been recognized to exhibit powerful anti-inflammatory and immunomodulatory effects [21]. Previous studies have shown that HO-1-inducing agents, as hemin, can mitigate nephrotoxic effects caused by a wide array of stressors, including mercury [15] and acetaminophen [20]. Based on the previous information, the present study aimed to examine whether the activation of HO-1 (by hemin) would have protective effects against CP induced nephrotoxicity in rats. For this purpose, we have evaluated the status of renal lipid peroxidative assay and antioxidant defenses. In addition, detailed glomerular and tubular pathologies were assessed.

## 2. Materials and Methods

### 2.1. Drugs and Chemicals

CP and hemin (powder) were purchased from Sigma Chemical Company, USA. Other chemical reagents were of high-quality analytical grade. Hemin was first dissolved in 0.1 M NaOH, titrated to pH 7.4 with 0.1 M HCl, and then diluted with normal saline (1 : 10 v/v), while CP was prepared in normal saline.

### 2.2. Animals and Treatments

Healthy adult male rats (110–140 g) were obtained from animal house facility at King Saud University, Saudi Arabia. Rats were housed in polyethylene cages under controlled laboratory conditions and provided with standard rat chow and water* ad libitum*. They were allowed 1 week of acclimatization before the initiation of the experiment. Experimental protocol of this study complies with the NIH ethical guidelines for the manipulation and care of laboratory animals. Rats were randomly assigned into 4 groups (*n* = 6):

- saline group (control): rats received 3 mL/kg 0.09% NaCl, intraperitoneally (i.p.);
- CP group: rats received a single dose of CP, i.p. (10 mg/kg);
- CP+hemin group: rats received 40 *μ*mol/kg hemin, subcutaneously (s.c.), 1 h following CP;
- hemin group: rats received 40 *μ*mol/kg hemin, s.c.

Doses, duration, and routes of exposure were chosen according to previously published reports [20, 22]. All rats were sacrificed under light ether anesthesia after 24 h of the last dose of specific treatment, and samples of trunk blood and kidneys were collected. Blood was centrifuged at 5000 rpm for 10 min and the separated sera were used for measurement of renal function tests. Kidneys were decapsulated and washed in cold isotonic saline. The cortex was carefully separated from medulla as described earlier by Banday et al. [23]. The kidney cortex was homogenized (Glass Col homogenizer) and a 20% w/v homogenate was prepared in ice cold 50 mM, pH 7.4 phosphate buffer saline. The homogenate was centrifuged at 5000 rpm for 20 min and the supernatant was then saved in aliquots to avoid sample thawing and freezing and stored at −80°C till used for assaying peroxidative damage and antioxidant status. Samples of the intact kidney tissues were used for light and electron microscopic studies.

### 2.3. Markers of Renal Toxicity

Serum levels of urea and creatinine were measured spectrophotometrically using commercial diagnostic kits (Human Gesellschaft für Biochemica und Diagnostica mbH, Germany), according to the methods described by Tabacco et al. [24] and Bartels and Böhmer [25], respectively.

### 2.4. Oxidative Stress-Related Indices

Malondialdehyde (MDA), an index of fatty acid oxidation, was estimated in quantifiable amounts using Thiobarbituric Acid Reactive Substances (TBARS) assay kit (BioAssay Systems, CA, USA) according to the method of Ohkawa et al. [26]. In this procedure, MDA reacts with thiobarbituric acid (TBA) to form a pink-colored complex that has maximum absorbance at 532 nm. MDA value was calculated in terms of nmol/g wet tissue. Nitric oxide (NO) level was determined in kidney homogenates using Nitrate/Nitrite Colorimetric Assay Kit (BioAssay Systems, CA, USA) according to the manufacturer's instructions. NO production was measured following reduction of nitrate to nitrite using improved Griess method [27]. Total NO synthetase (NOS) activity was detected by NOS assay kit (BioAssay Systems, CA, USA) [28]. Activities of glutathione peroxidase (GPx, EC 1.11.1.9) and superoxide dismutase (SOD, EC 1.15.1.1) as well as the level of reduced glutathione (GSH) in renal cortex were determined spectrophotometrically, according to the standard detection protocol of analysis kits (BioAssay Systems, CA, USA) [29–31]. Glutathione reductase (GR, EC 1.6.4.2) activity was assayed using a commercial kit from Cayman Chemical Company, USA [32]. Protein content was estimated by the method of Lowry et al. [33].

### 2.5. Light Microscopy

Kidney taken from each animal was fixed in 10% formalin solution, dehydrated in ascending series of ethanol, and embedded in paraffin. Sections (4 *μ*m-thick) were cut, stained with haematoxylin and eosin solutions, and examined under light microscope (Nikon 80i, Japan).

### 2.6. Electron Microscopy

Small slices of kidney cortex (*n* = 3 per group) were fixed in 3% glutaraldehyde in sodium phosphate buffer (200 mM, pH 7.2) for 3 h at 4°C. Postfixation was in cold 1% osmium tetroxide (Agar Sci. Ltd., England) for 1 h. After flushing in phosphate buffer, the tissue samples were dehydrated in graded ethanol solutions and embedded in Araldite (Agar Sci. Ltd., England). Ultrathin sectioning (80–100 nm) was carried out using Leica EM UC6 (Leica Co., Austria) ultramicrotome. Sections were mounted on grids, double stained with 2% uranyl acetate and lead citrate, and viewed under Jeol JEM 1011 transmission electron microscope (Jeol Ltd., Japan) at 80 kV.

### 2.7. Statistics

All variables were compared using one-way analysis of variance (ANOVA) followed by LSD multiple range test. Differences at *P* < 0.05 were considered significant. Statistical tests were performed using SAS statistical software (SAS v. 9.2, SAS Institute, Inc., Cary, NC). Data were presented as mean ± standard error (SE).

## 3. Results

### 3.1. Biochemical Findings

The results of biochemical analysis in all studied groups are shown in Table 1. Treatment of male rats with CP resulted in significant increases in levels of blood urea (3.1-fold) and creatinine (6.3-fold) compared to control animals, indicating renal damage. These changes were significantly reversed by hemin treatment. Hemin administration alone had no effect on kidney function. With respect to TBARS, renal levels were significantly higher in CP group than the control values, while TBARS levels were similar in CP plus hemin and control groups. The levels of NO in cortical tissue were significantly increased in rats injected with a single dose of CP as compared to control animals. Hemin treatment significantly decreased the elevated renal NO levels. In a similar manner, an elevation in NOS activity (4.6-fold) was observed in CP group compared to control group. In rats treated with hemin plus CP, the activity of NOS was decreased by 36.2% compared to CP-treated animals. On the other hand, the activities of GPx and GR were profoundly declined by approximately 30% in cortical homogenates of CP group compared to the corresponding control values. Administration of hemin prevented CP elicited decreases in activities of theses antioxidant enzymes. On the contrary, no statistically significant changes in SOD activity and GSH level were recorded after CP treatment; however, hemin administration either alone or in combination of CP enhanced renal GSH levels compared to the control group.

### 3.2. Kidney Histopathology

**Figure 1:**
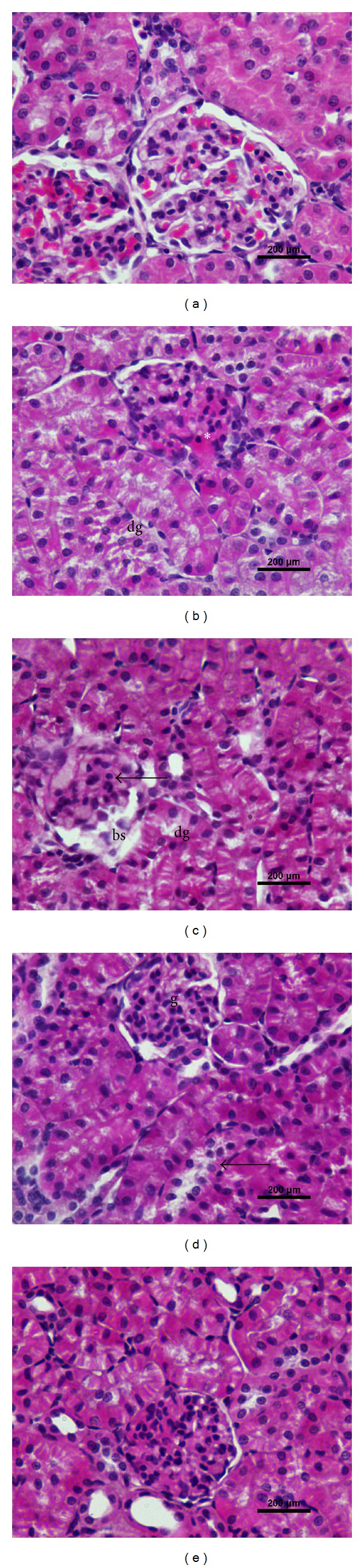
Photomicrographs of rat kidney. (a) Control group: normal histological structure. ((b)-(c)) CP group: severe glomerular congestion (∗, in (b)), glomerular atrophy (arrow, in (c)) and dilatation of Bowman's space (bs, in (c)), and degeneration of renal tubular cells (dg, in (b) and (c)). (d) CP plus hemin group: mild tubular degeneration (arrow), a normal-looking glomerulus (g) is also discernible. (e) Hemin group: renal histology is comparable to control.

Representative light micrographs of renal cortex are illustrated in Figure 1. The histological picture of kidney was normal in both control (Figure 1(a)) and hemin-treated (Figure 1(e)) groups. In CP-treated rats (Figures 1(b)-1(c)), the renal corpuscles displayed extensive congestion filling up the glomerular capillary loops. Some glomeruli were atrophied or lost with concurrent dilatation of Bowman's space. The morphological deterioration was also characterized by a widespread tubular cell swelling, necrosis, and degeneration, occurring primarily in proximal convoluted tubules (PCTs) epithelia. In addition, peritubular inflammatory cell infiltrations and hemorrhagic foci were clearly apparent after CP treatment. In contrast, these histological abnormalities were found to be reduced in CP plus hemin-treated rats (Figure 1(d)).  Table 2 shows a semiquantitative analysis of renal lesions in rats treated with CP with or without hemin.

### 3.3. Electron Microscopic Observations

**Figure 2:**
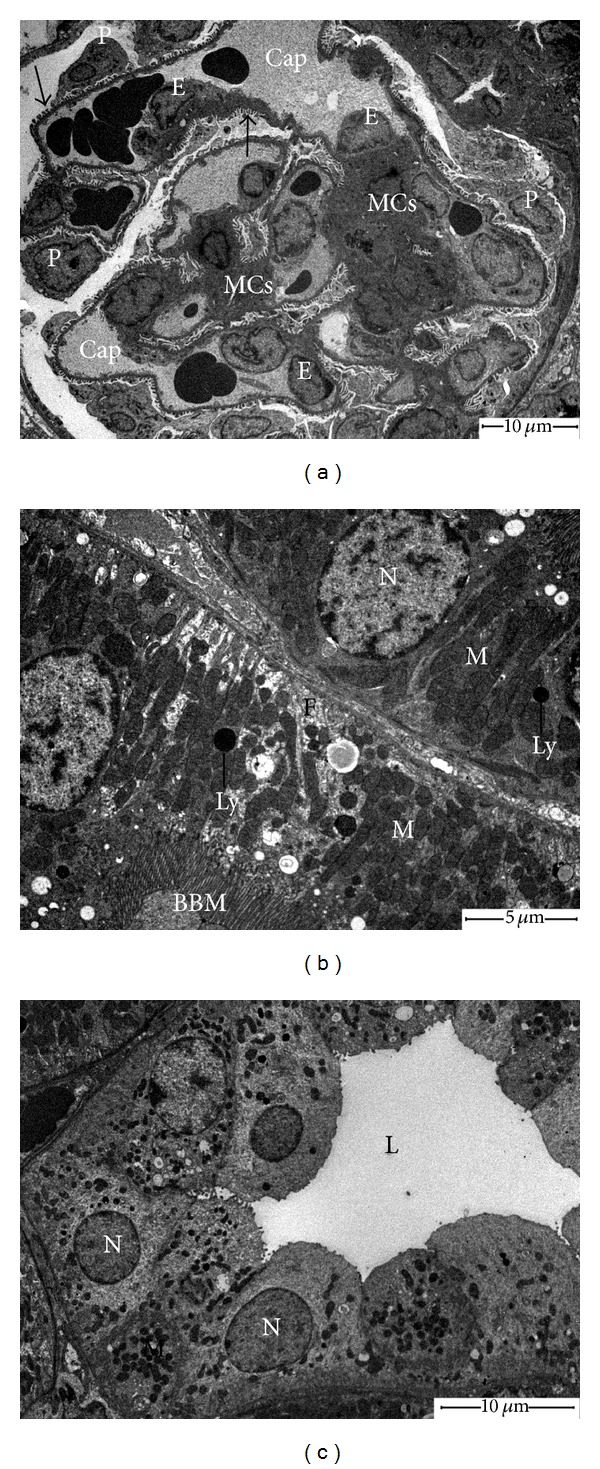
Electron micrographs of kidney of control rats. (a) Note the glomerulus has podocytes (P) which remain in contact with GBM by foot processes (arrows). The glomerular capillaries (Cap) are lined by endothelial cells (E) which are richly fenestrated and supported by mesangial cells (MCs) with their surrounding extracellular matrix. (b) Proximal tubules have well-developed brush border microvilli (BBM), and their lining epithelia contain large number of mitochondria (with normal feature) (M), few lysosomes (Ly), and many basal infoldings (F), N: nucleus. (c) Epithelial lining of distal tubule with apical nuclei (N), numerous mitochondria (M), short cisternae of rough endoplasmic reticulum, and few microvilli toward the lumen (L).

**Figure 3:**
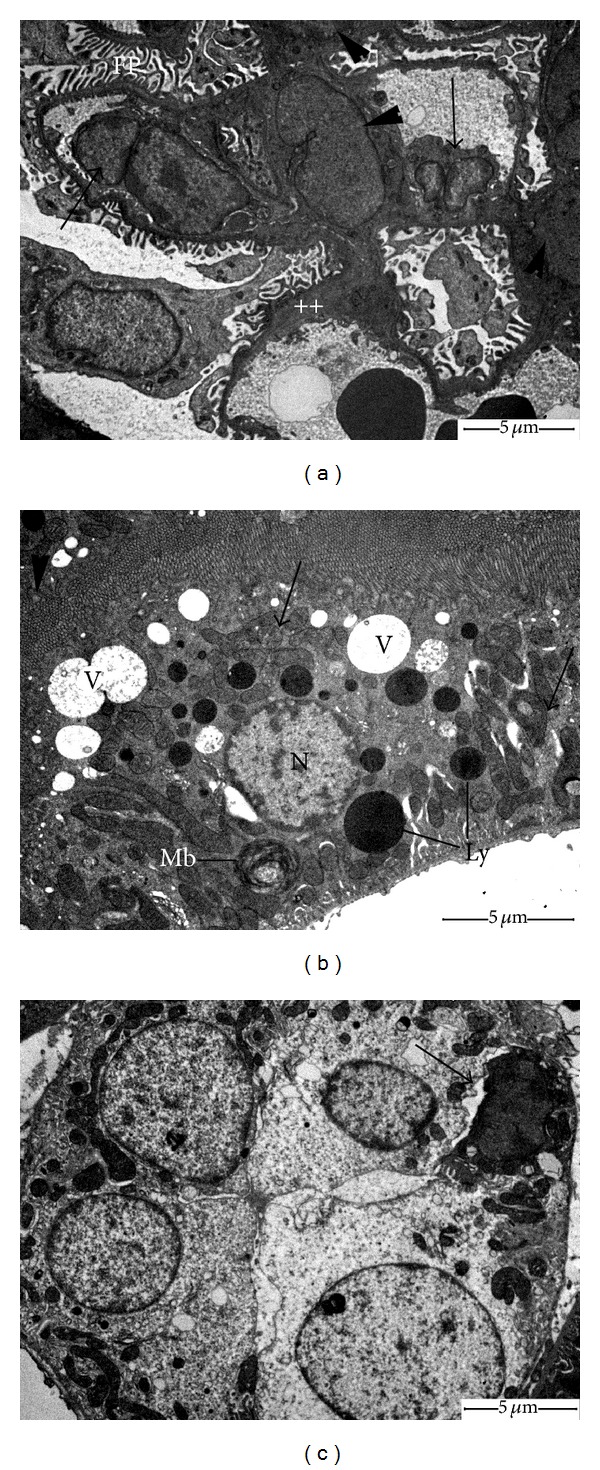
Electron micrographs of kidney of CP-treated rats. (a) Renal glomerulus with thickened GBM (++), capillary lumens are obliterated by endocapillary hypercellularity and hypertrophy (arrows). Note also fusion of secondary foot processes (FP) and mesangial hypercellularity (arrowheads). (b) Proximal convoluted tubule cell containing large cytoplasmic vacuoles (V), clusters of deformed mitochondria (arrows), numerous lysosomal bodies (Ly), and lamellar (myeloid) body (Mb). Observe nucleus (N) with condensed chromatin pattern. Arrowhead points to partial destruction of the brush border. (c) Abnormal distal tubular cells with few organelles. Arrow indicates apoptotic cell.

**Figure 4:**
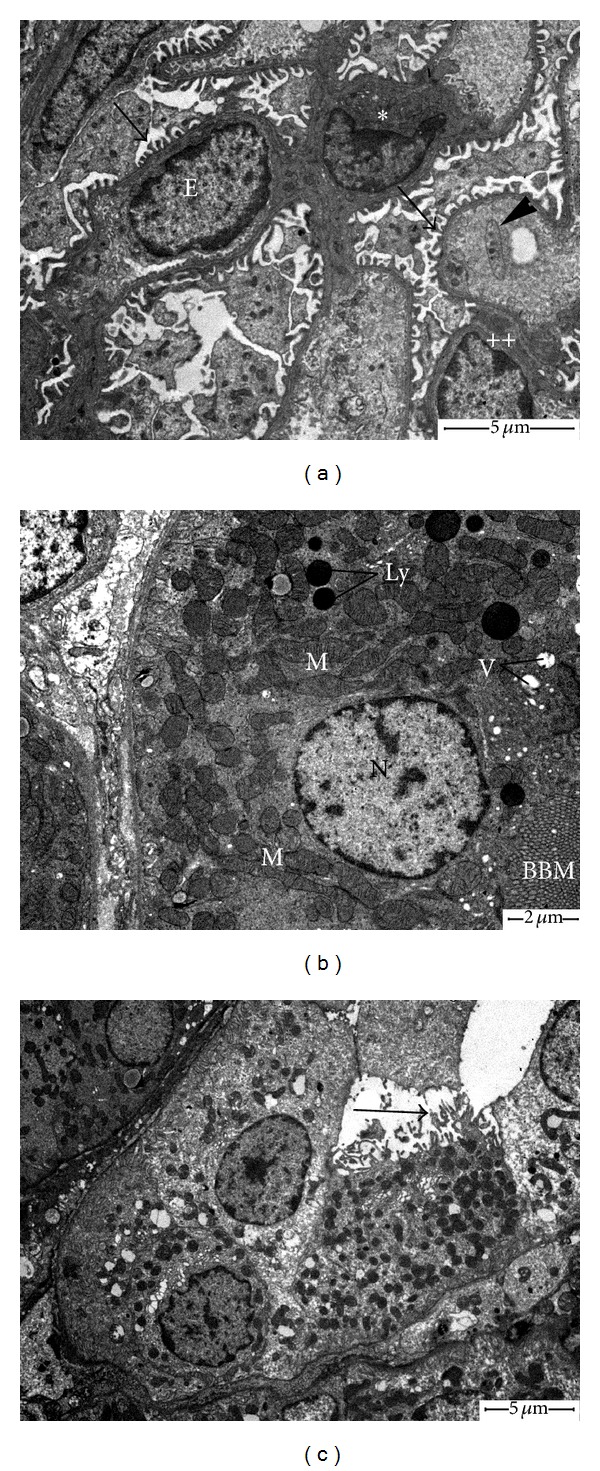
Electron micrographs of kidney of CP plus hemin-treated rats. (a) Glomerulus with nearly regular foot processes (arrows) and limited mesangial matrix (∗). Arrowhead indicates a resting platelet inside glomerular capillary vessel. Capillary endothelium (E) and slight thickening of GBM (++) are indicated. (b) Proximal convoluted tubule cell showing loss of large vacuolar structures; only small vacuoles (V) are seen beneath the intact brush border microvilli (BBM). Nucleus (N), mitochondria (M), and lysosomes are of normal morphology. (c) Dilated distal tubule with normal cellular ultrastructure. Detachment/loss of pleomorphic microvilli is indicated by arrow.

Renal cortical tissues of control animals revealed normal appearance of ultrastructural patterns of renal corpuscles and tubules (Figures 2(a)–2(c)). Obvious ultrastructural changes were noted in the glomeruli of CP-treated rats (Figure 3(a)). We observed progressive deformation of capillary endothelial cells, including hypertrophy (i.e., swelling), hyperplasia, and focal loss of fenestrae. The secondary foot processes of podocytes were deteriorated and markedly fused in some places. The Glomerular Basement Membrane (GBM) was corrugated and displayed uneven thickening. The mesangium was expanded with massive increase in matrix. Examination of PCTs of CP group revealed the presence of destructed/irregular microvilli, apical vacuoles, large secondary lysosomes, altered mitochondria, and accumulation of myeloid bodies in the cytoplasm (Figure 3(b)). The distal convoluted tubules (DCTs) epithelia of CP-treated rats lost most of their cytoplasmic electron density and cellular organelles (Figure 3(c)). Apoptotic cells were also seen and identified by cell shrinkage, degradation of chromatin, and membrane blebs. CP plus hemin group showed marked recovery in the ultrastructural aspect of glomeruli and cellular features of renal tubules (Figures 4(a)–4(c)).

## 4. Discussion

Kidneys are dynamic organs and represent one of the major homeostasis body systems; they are affected by diverse varieties of chemicals and drugs [34–36]. Platinum complexes (such as CP) are highly nephrotoxic, which predominantly accumulate in the kidneys [37]. It is evident from the present study that administration of a single dose of CP (7.5 mg/kg i.p.) resulted in acute tubule cell necrosis, interstitial inflammation, and glomerular congestion and atrophy. Besides, renal function biochemical parameters such as blood urea and creatinine were markedly elevated in CP-treated rats compared to control group, reflecting early damage of the filtration barrier (i.e., the fenestrated capillary endothelium, GBM, and visceral epithelial podocytes). These results are in agreement with previous reports in murine models [37, 38]. Hemin significantly suppressed the increases in blood urea and serum creatinine, which may be due to improvement in glomerular filtration damage induced by CP.

Development of therapies to prevent free radicals generation may influence renal oxidative damage and protect against irreversible cell damage and necrosis induced by CP. Excessive ROS production by CP causes antioxidant imbalance and leads to progressive LPO and antioxidant depletion. LPO adducts were previously shown to mediate glomerular lesions [39]. In addition, peroxidation of membrane phospholipids increases membrane fluidity and permeability and results in hypertrophy (i.e., swelling) of renal tubule cells and degeneration of cell organelles, such as nuclei and mitochondria [40]. ROS production is further stimulated by damaged mitochondria in CP nephrotoxicity [8]. Our results showed that CP induced a significant elevation in MDA (a secondary product of LPO) and decrease in activities of GPx and GR, which are in agreement with previous studies [11, 41–44]. In line with these data, the formation of large myeloid bodies (i.e., multilamellated lysosomes), as observed in PCTs epithelia of CP group, has been a hallmark alteration indicative of an inhibited or altered function of intracytoplasmic enzymatic machinery [45]. The administration of hemin decreased or abolished kidney oxidative stress/LPO and increased antioxidant potentials. Hemin was reported to induce HO-1 protein which exerts cellular protection against renal toxicants [15]. HO-1 expression blocks injury pathways by catalyzing the breakdown of prooxidant heme into biliverdin (BV), carbon monoxide (CO), and ferrous iron (Fe^+2^)/ferritin. BV formed in this reaction is rapidly recycled by the action of biliverdin reductase into bilirubin (BR) which has a higher free radical-scavenging activity and antiapoptotic properties [46–49]. In addition, CO triggers the nuclear factor-erythroid 2-related factor 2 (Nrf2) to increase the expression and function of a battery of antioxidant enzymes [50]. Kim et al. [51] demonstrated that hemin substantially stimulates nuclear translocation of Nrf2 and its subsequent binding to antioxidant responsive element (ARE), a regulatory enhancer sequence in the promoter region of the involved genes.

Treatment with CP overdose induces nitrosative stress by NO and other nitrosylating agents and this was correlated with the expression of the inducible nitric oxide synthase (iNOS) protein [52]. In the current study, NO production in the CP-treated group was significantly higher than that in the control group; our results are consistent with the results obtained by Kart et al. [53], who have shown that there is strong immunoreactivity against iNOS in the liver tissue of the CP-treated group. Recent work of Chirino et al. [54] reported that the downregulation of iNOS expression reduced CP-induced renal damage and nitrosative stress. High NO levels exert toxicological effects by reacting with superoxide anion to generate short-lived but hyperactive peroxynitrite radical with subsequent nitration of protein tyrosine residues [55, 56]. Also, NO output depletes intracellular GSH, which increases susceptibility to oxidative stress and aggravates renal tissue damage [57, 58], especially for glomerular diseases (e.g., lupus nephritis) [59]. Hemin-mediated augmentation of HO-1 activity was proved to be efficient enough to reduce the NO-dependent pathological and inflammatory conditions [17, 60, 61]. Of note, HO-1 and its reaction product CO also suppress the expression of iNOS protein by preventing the activation of nuclear factor-kappa B (NF-*κ*B) which upregulates the transcription of the iNOS gene [17, 62, 63].

In conclusion, this study is the first to demonstrate the protective role of hemin (an HO-1 activator) against acute nephrotoxicity induced by CP. Treatment of hemin ameliorated renal ultrastructural changes and dysfunction, and this was associated with reduction of LPO, overstimulation of antioxidant capacity, and suppression of NO biosynthesis, the proinflammatory mediator. However, further preclinical studies are needed to verify whether coadministration of CP and hemin could affect the outcome of tumor chemotherapy.

## Figures and Tables

**Table 1:** The results of biochemical analysis in control and experimental groups^a^.

Parameters	Unit	Control	CP	CP + hemin	Hemin
Urea	mg/dL	48.60 ± 1.50	151.61 ± 10.02^b^	86.61 ± 3.02^b,c^	53.60 ± 3.20^c^
Creatinine	mg/dL	0.81 ± 0.11	5.14 ± 0.76^b^	1.45 ± 0.69^c^	0.78 ± 0.08^c^
TBARS	nmole MDA/g tissue	6.44 ± 1.67	11.00 ± 1.05^b^	7.26 ± 2.74	4.06 ± 1.53^c^
NO	*µ*mole/g tissue	16.42 ± 0.98	19.58 ± 1.50^b^	13.16 ± 0.71^b,c^	11.86 ± 0.84^b,c^
NOS	nmole/g tissue	5.56 ± 0.84	25.61 ± 7.50^b^	16.34 ± 2.19	6.07 ± 0.89^c^
GPx	mU/mg protein	353.51 ± 44.43	238.49 ± 40.83^b^	311.15 ± 25.76	376.81 ± 30.18^c^
SOD	U/mg protein	18.14 ± 2.76	14.01 ± 1.85	17.59 ± 1.83	18.08 ± 1.15
GR	mU/mg protein	98.79 ± 8.40	67.63 ± 6.03^b^	86.26 ± 9.13	105.75 ± 10.73^c^
GSH	*µ*mole/mg protein	6.68 ± 1.10	6.93 ± 0.59	9.15 ± 1.01^b^	10.05 ± 0.37^b,c^

TBARS: thiobarbituric acid reactive substances, MDA: malondialdehyde, NO: nitric oxide, NOS: nitric oxide synthetase, GPx: glutathione peroxidase, SOD: superoxide dismutase, GR: Glutathione reductase, and GSH: reduced glutathione.

^a^Results are expressed as mean ± SE for six replicates.

^b^Significantly different from control at *P* < 0.05 by one-way ANOVA.

^c^Significantly different from CP at *P* < 0.05 by one-way ANOVA.

**Table 2:** Semiquantitative scoring of glomerular and tubulointerstitial lesions in control and experimental rats.

Group	Control	CP	CP + hemin	Hemin
Glomerular congestion	—	++	+	—
Glomerular atrophy	—	++	+	—
Peritubular inflammatory cell infiltration	—	+++	+	—
Tubular damage	—	+++	+	—

Scoring scale: none (—), mild (+), moderate (++), and severe (+++).

## References

[1] Atasayar S, Gürer-Orhan H, Orhan H, Gürel B, Girgin G, Özgüneş H (2009). Preventive effect of aminoguanidine compared to vitamin E and C on cisplatin-induced nephrotoxicity in rats. *Experimental and Toxicologic Pathology*.

[2] Hassan I, Chibber S, Naseem I (2010). Ameliorative effect of riboflavin on the cisplatin induced nephrotoxicity and hepatotoxicity under photoillumination. *Food and Chemical Toxicology*.

[3] Naqshbandi A, Khan MW, Rizwan S, Rehman SU, Khan F (2012). Studies on the protective effect of dietary fish oil on cisplatin induced nephrotoxicity in rats. *Food and Chemical Toxicology*.

[4] Rodrigues MAC, Rodrigues JL, Martins NM (2011). Carvedilol protects against cisplatin-induced oxidative stress, redox state unbalance and apoptosis in rat kidney mitochondria. *Chemico-Biological Interactions*.

[5] Antunes LMG, Darin JDC, de Lourdes P. Bianchi M (2000). Protective effects of vitamin C against cisplatin-induced nephrotoxicity and lipid peroxidation in adult rats: a dose-dependent study. *Pharmacological Research*.

[6] Badary OA, Abdel-Maksoud S, Ahmed WA, Owieda GH (2005). Naringenin attenuates cisplatin nephrotoxicity in rats. *Life Sciences*.

[7] Francescato HDC, Costa RS, Scavone C, Coimbra TM (2007). Parthenolide reduces cisplatin-induced renal damage. *Toxicology*.

[8] Yao X, Panichpisal K, Kurtzman N, Nugent K (2007). Cisplatin nephrotoxicity: a review. *The American Journal of the Medical Sciences*.

[9] Chirino YI, Sánchez-González DJ, Martínez-Martínez CM, Cruz C, Pedraza-Chaverri J (2008). Protective effects of apocynin against cisplatin-induced oxidative stress and nephrotoxicity. *Toxicology*.

[10] Pérez-Rojas JM, Guerrero-Beltrán CE, Cruz C, Sánchez-González DJ, Martínez-Martínez CM, Pedraza-Chaverri J (2011). Preventive effect of tert-butylhydroquinone on cisplatin-induced nephrotoxicity in rats. *Food and Chemical Toxicology*.

[11] Domitrović R, Cvijanović O, Pernjak-Pugel E, Škoda M, Mikelić L, Crnčević-Orlić Ž (2013). Berberine exerts nephroprotective effect against cisplatin-induced kidney damage through inhibition of oxidative/nitrosative stress, inflammation, autophagy and apoptosis. *Food and Chemical Toxicology*.

[12] Pan H, Shen K, Wang X, Meng H, Wang C, Jin B (2014). Protective effect of metalloporphyrins against Cisplatin-induced kidney injury in mice. *PLoS ONE*.

[13] Ali BH, Al Moundhri MS (2006). Agents ameliorating or augmenting the nephrotoxicity of cisplatin and other platinum compounds: a review of some recent research. *Food and Chemical Toxicology*.

[14] Kim HP, Pae H-O, Back SH (2011). Heme oxygenase-1 comes back to endoplasmic reticulum. *Biochemical and Biophysical Research Communications*.

[15] Yoneya R, Ozasa H, Nagashima Y (2000). Hemin pretreatment ameliorates aspects of the nephropathy induced by mercuric chloride in the rat. *Toxicology Letters*.

[16] Chiu H, Brittingham JA, Laskin DL (2002). Differential induction of heme oxygenase-1 in macrophages and hepatocytes during acetaminophen-induced hepatotoxicity in the rat: effects of hemin and biliverdin. *Toxicology and Applied Pharmacology*.

[17] Wen T, Wu Z-M, Liu Y, Tan Y-F, Ren F, Wu H (2007). Upregulation of heme oxygenase-1 with hemin prevents d-galactosamine and lipopolysaccharide-induced acute hepatic injury in rats. *Toxicology*.

[18] Chen Y-S, Zhu X-X, Zhao X-Y, Xing H-Y, Li Y-G (2008). Hemin, a heme oxygenase-1 inducer, improves aortic endothelial dysfunction in insulin resistant rats. *Chinese Medical Journal*.

[19] Fouad AA, Qureshi HA, Al-Sultan AI, Yacoubi MT, Ali AA (2009). Protective effect of hemin against cadmium-induced testicular damage in rats. *Toxicology*.

[20] Fouad AA, Yacoubi MT, El-Bidawy MH (2009). Therapeutic potential of hemin in acetaminophen nephrotoxicity in rats. *Environmental Toxicology and Pharmacology*.

[21] Origassa CST, Câmara NOS (2013). Cytoprotective role of heme oxygenase-1 and heme degradation derived end products in liver injury. *World Journal of Hepatology*.

[22] Hussein A, Ahmed AAE, Shouman SA, Sharawy S (2012). Ameliorating effect of DL-α-lipoic acid against cisplatin-induced nephrotoxicity and cardiotoxicity in experimental animals. *Drug Discoveries & Therapeutics*.

[23] Banday AA, Farooq N, Priyamvada S, Yusufi ANK, Khan F (2008). Time dependent effects of gentamicin on the enzymes of carbohydrate metabolism, brush border membrane and oxidative stress in rat kidney tissues. *Life Sciences*.

[24] Tabacco A, Meiattini F, Moda E, Tarli P (1979). Simplified enzymic/colorimetric serum urea nitrogen determination. *Clinical Chemistry*.

[25] Bartels H, Böhmer M (1971). Micro-determination of Creatinine. *Clinica Chimica Acta*.

[26] Ohkawa H, Ohishi N, Yagi K (1979). Assay for lipid peroxides in animal tissues by thiobarbituric acid reaction. *Analytical Biochemistry*.

[27] Bulau P, Zakrzewicz D, Kitowska K (2007). Analysis of methylarginine metabolism in the cardiovascular system identifies the lung as a major source of ADMA. *American Journal of Physiology—Lung Cellular and Molecular Physiology*.

[28] Ghigo D, Riganti C, Gazzano E, Costamagna C, Bosia A (2006). Cycling of NADPH by glucose 6-phosphate dehydrogenase optimizes the spectrophotometric assay of nitric oxide synthase activity in cell lysates. *Nitric Oxide—Biology and Chemistry*.

[29] Jacobson B, Quigley G, Lockitch G (1988). Adaptation of glutathione peroxidase assay to the Technicon RA-1000. *Clinical Chemistry*.

[30] Ukeda H, Maeda S, Ishii T, Sawamura M (1997). Spectrophotometric assay for superoxide dismutase based on tetrazolium salt 3'-{1-[(phenylamino)-carbonyl]-3,4-tetrazolium}-bis(4-methoxy-6- nitro)benzenesulfonic acid hydrate reduction by xanthine-xanthine oxidase. *Analytical Biochemistry*.

[31] Carlberg I, Mannervik B (1985). Glutathione reductase. *Methods in Enzymology*.

[32] Lindenmaier H, Becker M, Haefeli WE, Weiss J (2005). Interaction of progestins with the human multidrug resistance-associated protein 2 (MRP2). *Drug Metabolism and Disposition*.

[33] Lowry OH, Rosebrough NJ, Farr AL, Randall RJ (1951). Protein measurement with the Folin phenol reagent. *The Journal of biological chemistry*.

[34] Abdel-Moneim AM, Said KM (2007). Acute effect of cadmium treatment on the kidney of rats: biochemical and ultrastructural studies. *Pakistan Journal of Biological Sciences*.

[35] Al Kahtani MA, Abdel-Moneim AM, El-Sayed WM (2014). The influence of taurine pretreatment on aluminum chloride induced nephrotoxicity in Swiss albino mice. *Histology and Histopathology*.

[36] Salem NA, Salem EA (2011). Renoprotective effect of grape seed extract against oxidative stress induced by gentamicin and hypercholesterolemia in rats. *Renal Failure*.

[37] Abdelmegmd NE, Chmaisseand HN, Abou Zeinab NS (2010). Silymarin ameliorates Cisplatin-induced hepatotoxicity in rats: histopathological and ultrastructural studies. *Pakistan Journal of Biological Sciences*.

[38] Morigi M, Imberti B, Zoja C (2004). Mesenchymal stem cells are renotropic, helping to repair the kidney and improve function in acute renal failure. *Journal of the American Society of Nephrology*.

[39] Binder CJ, Weiher H, Exner M, Kerjaschki D (1999). Glomerular overproduction of oxygen radicals in Mpv17 gene-inactivated mice causes podocyte foot process flattening and proteinuria. A model of steroid-resistant nephrosis sensitive to radical scavenger therapy. *The American Journal of Pathology*.

[40] Foulkes EC (1988). *Biological Membranes in Toxicology*.

[41] Sueishi K, Mishima K, Makino K (2002). Protection by a radical scavenger edaravone against cisplatin-induced nephrotoxicity in rats. *European Journal of Pharmacology*.

[42] Işeri S, Ercan F, Gedik N, Yüksel M, Alican I (2007). Simvastatin attenuates cisplatin-induced kidney and liver damage in rats. *Toxicology*.

[43] Ekor M, Emerole GO, Farombi EO (2010). Phenolic extract of soybean (*Glycine max*) attenuates cisplatin-induced nephrotoxicity in rats. *Food and Chemical Toxicology*.

[44] Li Y-N, Guo Y, Xi M-M (2014). Saponins from *Aralia taibaiensis* attenuate D-galactose-induced aging in rats by activating FOXO3a and Nrf2 pathways. *Oxidative Medicine and Cellular Longevity*.

[45] Hermenean A, Ardelean A, Stan M (2013). Protective effects of naringenin on carbon tetrachloride-induced acute nephrotoxicity in mouse kidney. *Chemico-Biological Interactions*.

[46] Barañano DE, Rao M, Ferris CD, Snyder SH (2002). Biliverdin reductase: a major physiologic cytoprotectant. *Proceedings of the National Academy of Sciences of the United States of America*.

[47] Abraham NG, Cao J, Sacerdoti D, Li X, Drummond G (2009). Heme oxygenase: the key to renal function regulation. *American Journal of Physiology: Renal Physiology*.

[48] Ryter SW, Choi AM (2010). Heme oxygenase-1/carbon monoxide: novel therapeutic strategies in critical care medicine. *Current Drug Targets*.

[49] Naito Y, Takagi T, Uchiyama K, Yoshikawa T (2011). Heme oxygenase-1: a novel therapeutic target for gastrointestinal diseases. *Journal of Clinical Biochemistry and Nutrition*.

[50] Chan K, Han X-D, Kan YW (2001). An important function of Nrf2 in combating oxidative stress: detoxification of acetaminophen. *Proceedings of the National Academy of Sciences of the United States of America*.

[51] Kim Y-C, Masutani H, Yamaguchi Y, Itoh K, Yamamoto M, Yodoi J (2001). Hemin-induced activation of the thioredoxin gene by Nrf2: a differential regulation of the antioxidant responsive element by a switch of its binding factors. *The Journal of Biological Chemistry*.

[52] Curran RD, Ferrari FK, Kispert PH (1991). Nitric oxide and nitric oxide-generating compounds inhibit hepatocyte protein synthesis. *The FASEB Journal*.

[53] Kart A, Cigremis Y, Karaman M, Ozen H (2010). Caffeic acid phenethyl ester (CAPE) ameliorates cisplatin-induced hepatotoxicity in rabbit. *Experimental and Toxicologic Pathology*.

[54] Chirino YI, Trujillo J, Sánchez-González DJ (2008). Selective iNOS inhibition reduces renal damage induced by cisplatin. *Toxicology Letters*.

[55] Radi R, Peluffo G, Alvarez MN, Naviliat M, Cayota A (2001). Unraveling peroxynitrite formation in biological systems. *Free Radical Biology and Medicine*.

[56] Pacher P, Beckman JS, Liaudet L (2007). Nitric oxide and peroxynitrite in health and disease. *Physiological Reviews*.

[57] Clancy RM, Abramson SB (1995). Nitric oxide: a novel mediator of inflammation. *Proceedings of the Society for Experimental Biology and Medicine*.

[58] Liu P, Hock CE, Nagele R, Wong PY (1997). Formation of nitric oxide, superoxide, and peroxynitrite in myocardial ischemia-reperfusion injury in rats. *The American Journal of Physiology*.

[59] Takeda Y, Takeno M, Iwasaki M (2004). Chemical induction of HO-1 suppresses lupus nephritis by reducing local iNOS expression and synthesis of anti-dsDNA antibody. *Clinical and Experimental Immunology*.

[60] Choi B-M, Pae H-O, Kim Y-M, Chung H-T (2003). Nitric oxide-mediated cytoprotection of hepatocytes from glucose deprivation-induced cytotoxicity: involvement of heme oxygenase-1. *Hepatology*.

[61] Yang S, Shih H-J, Chow Y-C (2007). The protective role of heme oxygenase-1 induction on testicular tissues after testicular torsion and detorsion. *The Journal of Urology*.

[62] Ashino T, Yamanaka R, Yamamoto M (2008). Negative feedback regulation of lipopolysaccharide-induced inducible nitric oxide synthase gene expression by heme oxygenase-1 induction in macrophages. *Molecular Immunology*.

[63] Taye A, Ibrahim BM (2013). Activation of renal haeme oxygenase-1 alleviates gentamicin-induced acute nephrotoxicity in rats. *Journal of Pharmacy and Pharmacology*.

